# Functional and Molecular Markers for Hearing Loss and Vertigo Attacks in Meniere’s Disease

**DOI:** 10.3390/ijms24032504

**Published:** 2023-01-28

**Authors:** Chao-Hui Yang, Ming-Yu Yang, Chung-Feng Hwang, Kuang-Hsu Lien

**Affiliations:** 1 Department of Otolaryngology-Head & Neck Surgery, Kaohsiung Chang Gung Memorial Hospital and Chang Gung University College of Medicine, Kaohsiung 83301, Taiwan; 2 Graduate Institute of Clinical Medical Sciences, College of Medicine, Chang Gung University, Taoyuan 33302, Taiwan; 3 Department of Otolaryngology-Head & Neck Surgery, Chang Gung Memorial Hospital, Linkou Branch, College of Medicine, Chang Gung University, Taoyuan 33302, Taiwan

**Keywords:** Meniere’s disease, markers, pure tone audiometry, electrocochleography, vestibular evoked myogenic potential, caloric test, video head impulse test, magnetic resonance imaging, autoimmunity, vasopressin, circadian clock, vestibular migraine, hearing loss, vertigo attacks

## Abstract

Meniere’s disease (MD) is one of the most complicated diseases in the otologic clinic. The complexity of MD is partially due to the multifactorial etiological mechanisms and the heterogenous symptoms, including episodic vertigo, hearing loss, aural fullness and tinnitus. As a result, the diagnosis of MD and differentiating MD from other diseases with similar symptoms, such as vestibular migraine (VM), is challenging. In addition, it is difficult to predict the progression of hearing loss and the frequency of vertigo attacks. Detailed studies have revealed that functional markers, such as pure tone audiometry (PTA), electrocochleography (ECochG), vestibular evoked myogenic potential (VEMP), caloric test, video head impulse test (vHIT) and magnetic resonance imaging (MRI) could help to evaluate MD with different hearing levels and frequency of vertigo attacks. Investigations of molecular markers such as autoimmunity, inflammation, protein signatures, vasopressin and circadian clock genes in MD are still underway. This review will summarize these functional and molecular markers, address how these markers are associated with hearing loss and vertigo attacks in MD, and analyze the results of the markers between MD and VM.

## 1. Introduction

Meniere’s disease (MD) is a heterogeneous inner ear disorder with complex symptoms, including episodic vertigo, sensorineural hearing loss and aural symptoms such as aural fullness or tinnitus. The incidence and prevalence of MD are varied and range from 3.5 per 100,000 to 513 per 100,000 [1]. The most classic pathogenesis of MD is endolymphatic hydrops (EH). According to the published temporal bone finding from the histopathological studies, EH of pars inferior structures was found in 98.8–100% of all confirmed cases [2,3,4]. Different pathogeneses of MD were claimed in recent decades, including anatomic or structural changes, vasopressin, autoimmune, allergy, migraine-related, genetic theories, and so on [5,6,7,8,9,10,11,12,13,14,15]. In fact, it is a multifactorial disease with more than one etiology converging into characteristic symptomatology [16].

The clinical heterogeneity makes the diagnosis of MD a challenge in the clinic since we lack good objective markers and exact examination standards but only depend on subjective symptoms and signs. In 2015, the American Academy of Otolaryngology–Head and Neck Surgery Foundation (AAO-HNSF) revised the diagnostic criteria for two MD categories: Definite MD and probable MD (Table 1) [17,18]. These diagnostic criteria are important in diagnosing MD and distinguishing MD from other causes of vertigo with similar symptoms. However, because of the variable clinical presentation of MD, it needs to take a period of time to observe the clinical manifestations to make an accurate diagnosis of definite MD.

Fluctuating low-tone hearing loss is the most characteristic finding in early MD. It was hypothesized that the distention of basilar membranes in EH starts from the apex and causes low-frequency hearing loss [19]. Therefore, the severity of cochlear EH can be evaluated by the degree of hearing loss. Therefore, the most commonly used staging of MD is based on the average of pure tone thresholds at 0.5, 1, 2 and 3 kHz of the audiogram: stage I, less than 26 dB; stage II, 26–40 dB; stage III, 41 to 70 dB; stage IV, more than 70 dB [20]. In general, the history of hearing loss in MD is usually progressive but sometimes inconsistent between different subtypes of MD patients [21,22]. In addition, the frequency of vertigo attacks and vestibular hypofunction seem not able to predict the hearing outcome of MD [22,23]. However, no specific markers could be used to correlate with the hearing results of MD patients in the clinic.

The frequency of vertigo attacks is another concern for patients with MD since vertigo episodes could affect the quality of life. Although environmental factors might precipitate the attacks of MD, these factors are generally unspecific, and the factors that could induce vertigo in one patient may not affect another one. Therefore, the investigation for markers to predict vertigo attacks is essential for MD patients. On the other hand, clinicians need available markers to differentiate MD from vestibular migraine (VM), a disease comprising similar symptoms to MD. It is crucial since the prognosis and treatment strategies of MD and VM are different.

The diagnosis of MD is based on the diagnostic criteria of MD. Although several audiovestibular markers have been used to evaluate MD in the clinic, clinicians and patients are frequently confused with the results. In addition, there is no consensus regarding the role of molecular markers based on the etiological mechanisms of MD. In this review, we will overview the recent studies about the markers of functional testing used in the clinic and the recent development of magnetic resonance imaging (MRI) to evaluate MD. In addition, the molecular markers investigated in recent years will be reviewed. We will also focus on the functional and molecular markers in different hearing levels/staging and vertigo attacks/remission. In addition, we will look at the articles to see whether these markers could help to differentiate MD from VM.

## 2. Functional Markers for MD

### 2.1. Pure Tone Audiometry (PTA)

PTA is the most widely used hearing examination to identify hearing threshold levels and to determine the hearing loss degree, type and configuration. According to current AAO-HNS diagnostic criteria, one of the essential conditions of definite MD is the documented fluctuating low- to mid-frequency sensorineural hearing loss, which must be examined by the PTA [17]. Therefore, PTA is a necessary examination for suspected MD patients. In early MD presentation, hearing loss usually fluctuates or is subtle, making it difficult to catch a positive finding using PTA, and the suspected patient always undergoes several PTAs. In addition, the reliability and accuracy of PTA results depend on the cooperation of patients. However, PTA is still the basic test to accurately diagnose and determine the stage of MD.

During the natural course of MD, the hearing loss pattern could shift from the early stages of lower-frequency hearing loss to the late stage of high-frequency hearing loss, showing a “flat type audiogram” in PTA [23]. Therefore, PTA could be used in predicting the course of the disease. It was also observed that MD patients with middle- and high-frequency hearing loss at the initial visit had a poor prognosis in relation to hearing loss [21]. On the other hand, patients with primary MD exhibit moderate-to-severe hearing loss within 5–10 years, whereas patients with migraine-related MD or VM tend to recover and fluctuate for a long time [21,24].

Although a fluctuating sensorineural hearing loss, which affects low frequencies, is the major initial finding of PTA in MD, other similar diseases, such as acute low-tone hearing loss (ALHL), also characterizes similar PTAs. In particular, recurrent hearing loss is not uncommon in ALHL [25] and was previously thought to be “cochlear MD” [26]. Because of the clinical similarity of recurrent low-tone hearing loss with MD, one may wonder whether the recurrence of low-tone hearing loss is a sign of EH and whether it will progress to definite MD. In a study conducted by Yamasoba et al., the author followed patients with initial ALHL without vertigo for a minimum of 3 years, and only 11% developed clinical MD [27]. In a later study by Junicho et al., they also found that not all ALHL suffered from EH, even if they had a vertigo attack at the onset [28]. The reason is probably that recurrent ALHL might be a common sign of several diseases, such as MD, sudden deafness, and VM [29]. Recently, it was also observed that recurrent low-tone hearing loss possibly occurred in patients with migraine without vertigo, which was proposed as the symptoms of “cochlear migraine” [30]. Therefore, one cannot solely use PTA to diagnose MD, and definite MD has to be confirmed according to the diagnosis criteria.

In terms of vertigo attacks, fluctuating hearing loss is not always related to vestibular symptoms. Sometimes there is a time delay between hearing loss and vertigo [31]. During the natural course of the disease, some studies assume that the frequency of vertigo increases during the early stage of MD and may then keep stable or without vertigo attack for several years [32,33]. However, it was also observed that some patients with long-term MD still suffered from frequent vertigo attacks [34]. Compared to the progression of hearing loss in typical MD, the frequency of vertigo attacks is difficult to predict. The relationship between PTA and vertigo attacks during the course of MD needs further elucidation.

### 2.2. Electrocochleography (ECochG)

ECochG is an objective examination conducted to record the electrical potentials generated in the inner ear and auditory nerve in response to sound stimulation in order to detect the distortion of the basilar membrane due to EH [14]. Three main basic potentials in ECochG are the action potential (AP), the cochlear microphonics (CM) and the summating potential (SP). Since Gibson et al. proposed the abnormal findings of the ECochG in MD decades ago [35], several other studies showed that the amplitude of the AP and SP ratio could identify EH or MD. Although several researchers tried to use ECochG to diagnose MD, a standardized cut-off value to confirm EH is lacking. A recent systemic review revealed that the sensitivity of ECochG is about 66.7–85.7%, while the specificity is about 80–100% [36]. The problem of low sensitivity is possibly due to the fact that patients with probable MD may not have developed cochlear changes that result in an abnormal ECochG [37]. In addition, an elevated SP/AP ratio can be observed in other inner ear diseases, such as superior semicircular canal dehiscence [36]. In addition, the SP/AP ratio does not recover even if vertigo attacks disappear in MD [38]. Because the extratympanic electrode to measure the SP and AP ratio provided low specificity and sensitivity [39,40], transtympanic ECochG was developed to enhance the sensitivity [41,42]. Other methods to increase sensitivity include the usage of tone burst stimuli [43,44] and the measurement of the SP/AP area ratio [45], SP bias ratio [46], and graphic angle [47].

Since a positive ECochG represents EH, the correlation between ECochG results and the audiological symptoms of MD has been investigated. In the study conducted by Hornibrrok et al., the “positive” ECochG group had a significantly higher proportion of participants who showed asymmetrical hearing thresholds than the “negative” ECochG group [42]. In fact, a significant association between an enlarged SP/AP ratio and the degree of hearing loss was noted, showing that patients with advanced staging had a higher possibility of an enlarged SP/AP ratio [48]. Another study by Takeda et al. found that the incidence of an enhanced SP was increased in cases with MD with a pure-tone average exceeding 31 dB [49]. Therefore, they concluded that ECochG is more likely to be positive in patients with longer periods of cochlear and vestibular symptoms. However, in their study, the elevated SP/AP ratio may persist even in glycerol-induced hearing gain. This implied that even though ECochG might be used as the diagnostic tool in MD, the usefulness of ECochG as the marker for hearing loss in MD needs further investigation.

Another important topic is whether ECochG could differentiate MD from VM. Since EH is a distinct feature of MD, Mertines et al. observed a higher proportion of abnormal ECochG in MD than in VM [50]. However, EH still possibly occurred in patients of VM, which revealed a higher SP/AP [51]. Therefore, ECochG could not be used as the sole tool to differentiate MD and VM.

### 2.3. Vestibular Evoked Myogenic Potential (VEMP)

VEMP is a vestibular function technique used to evaluate the function of the utricle and saccule. Generally, VEMPs can be recorded from the contracted sternocleidomastoid muscle (cervical VEMPs or cVEMPs) to assess saccule function, while the inferior oblique muscle (ocular VEMPs or oVEMPs) to assess utricle function [52,53]. Air-conduction sound (ACS) and bone-conduction vibration (BCV) are the most frequently applied in clinical VEMP settings [54]. Compared to a healthy control, MD patients presented both cVEMP and oVEMP larger amplitudes when using BCV than ACS and both lower response rates when using ACS than BCV [55,56]. Another parameter of VEMP is the interaural amplitude difference (IAD) ratio. The higher ratio may indicate lower vestibular function [57]. In a meta-analysis, the sensitivity and specificity of cVEMP for identifying EH were 49% and 95% [58]. In fact, the evidence is insufficient to determine whether VEMP is useful for diagnosing MD. Therefore, VEMP might not be used as a reliable marker to diagnose MD but could serve as an adjuvant measurement of vestibular dysfunction [59] or as a component of the inner ear battery test for mapping the topographic involvement of EH in MD [56].

In addition to evaluating the inner ear function in MD, there are some implications of VEMP to help clinicians and MD patients. For example, since saccule is the second most frequent site of EH, VEMP was also used to assess the stage of MD. In a study conducted by Young et al., the IAD ratio of VEMP increased in the advanced stage of MD [57]. They then concluded that VEMP might provide another aid for evaluating the staging of MD in addition to the hearing test. Interestingly, because the saccule is spare in acute low-tone hearing loss, VEMP test may also be used to differentiate acute low-tone hearing loss from MD [60,61]. Additionally, the VEMP could also help predict vertigo attack frequency [62] and identify asymptomatic EH in the unaffected ear for evolving bilateral MD [63]. Of note, the result of VEMP could differ between quiescence and acute attack status [64]. Therefore, heterogeneous stages and disease status should be put into consideration during the interpretation of VEMP in MD patients.

In recent years, several studies attempted to use VEMP to differentiate MD from VM. Reduced click-evoked cVEMP and oVEMP amplitudes were observed in MD and VM compared to the control group [65,66]. However, it was found that the MD group showed reduced tone-evoked amplitudes for oVEMP [65] and a higher prevalence of increased IAD ratios compared to the VM group [66]. Additionally, the affected ears of MD had higher percentages of absent cVEMP and oVEMP responses [67]. Moreover, the IAD ratio in MD patients appeared to increase or remain stable over time, whereas VM patients showed fluctuating or stable IAD ratios. These studies implied that VEMP might be a potential functional marker for differentiating MD and VM, but more studies are needed to clarify this.

### 2.4. Caloric Test/Video Head Impulse Test (vHIT)

The caloric test and vHIT are frequently used to evaluate the vestibular function of the semicircular canal. In the caloric test, the horizontal semicircular canals can be stimulated via warm and cool water or air and then the change in endolymph gravity or thermal-induced pressure is expected [68,69] to reflect the low-frequency stimulation of the horizontal semicircular canal. It was noted that about 47–67% of patients with MD have unilateral canal weakness [70,71]. Caloric responses are usually diminished during the attacks of MD [72], and the incidence of canal paresis in the caloric test is higher in the advanced stage of MD [73]. In contrast, the abnormality rate of vHIT, a test that uses high-frequency stimulation to evaluate the function of six semicircular canals, varied between studies. The fluctuation of vestibulo–ocular reflex (VOR) function during and between acute vertigo attacks might explain the inconsistent results of vHIT among studies [72,74,75]. In addition, no differences in abnormal vHIT results between different stages and duration of MD were observed [73,76]. In general, the caloric test can detect vestibular abnormalities better than vHIT, and the discordance between the caloric test and vHIT was thought to be a marker for MD [70,77].

Since MD and VM share similar clinical features of vestibular symptoms, both diseases could exhibit abnormalities in the caloric test compared to healthy controls. However, the incidences of an abnormal caloric test are higher in MD than in VM [66,78]. Although horizontal VOR (hVOR) deficit could be found in VM patients, its incidence is lower than in MD [78,79]. It was suggested that vestibular testing with the caloric test still seems more sensitive for detecting hVOR pathology than vHIT when differentiating MD from VM [79].

### 2.5. MRI

Since EH is one of the most predominant histological demonstrations in MD, Nakashima et al. first proffered the visualization of EH after the intratympanic injection of gadolinium under MRI imaging in humans. MRI with three-dimensional fluid-attenuated inversion recovery (3D-FLAIR) is considered one of the most straightforward tools to recognize the EH directly in certain MD cases [80]. Later on, the same team demonstrated the EH in MD after the intravenous administration of gadolinium [81,82,83]. Recently, new“HYDROPS” (a hybrid of the reversed image of positive endolymph signal and native image of positive perilymph signal) and the advanced techniques after the intravenous administration of single-dose gadodiamide were claimed to improve the contrast in the production of a positive endolymph image and positive perilymph images [84]. Meanwhile, several MRI grading systems were used to quantitatively calibrate the enlargement of the endolymphatic spaces. For example, the three-stage system to record the perilymphatic and vestibular space and the four-stage system to include cochlear EH to ameliorate MD diagnosis [85,86] were proposed. Recently, significant correlations between the hearing level and the EH degree when using MRI were shown [87,88,89,90]. On the other side, there was no significant relationship between the extent or duration of vertigo and EH presentation when using MRI [87,90]. Although EH in MRI was associated with vertigo attacks in MD patients [87,88,89,91,92], the stability of EH was still observed during and after vertigo attacks [93]. As a result, the specific MRI stage correlated to hearing level and vertigo attacks in MD patients has not yet been well established but complemented MRI images can provide additional information to evaluate EH in MD patients [88,90,94,95,96,97,98].

Although the EH can be visualized in MRI and is considered a specific characteristic in MD, some studies found that EH can also be present in some VM patients [99,100,101,102,103]. Particularly, EH shown in MRI often correlated with auditory symptoms both in MD and VM [102]. However, a higher incidence of EH was observed in MD compared to VM [102,103]. On the other hand, Leng et al. reported the significance of the anatomical variations of these two diseases via non-contrast MRI, indicating that compared with the VM patients, patients with unilateral MD exhibited a shorter distance between the vertical part of the posterior semicircular canal and the posterior fossa with poorer vestibular aqueducts visibility in MRI [104]. However, a low diagnostic value was noted using these radiological variations. As such, the usage of MRI as a single functional image marker to differentiate MD and VM remains insufficient and needs further evidence.

### 2.6. The Problems and Future Directions of Functional Markers for MD

Although traditional markers such as ECochG/caloric tests have been used in MD diagnosis and evaluation for years, newer examinations such as VEMP/vHIT have recently been largely investigated. However, the relatively low sensitivity of these tools (particularly for the early stage of MD) is still a major problem for clinicians when using these tests as markers for diagnosing MD. In particular, the fluctuating features of MD symptoms might affect the results of functional markers. Therefore, the diagnosis of MD still needs to depend on the clinical diagnosis criteria. Further studies are necessary to evaluate the results according to different stages, duration of disease and vertigo attack phase to elucidate the role of functional markers in MD. So far, these tests could still provide complementary information to assess the vestibular function in MD patients in the clinic [105]. In recent years, MRI has become a cutting-edge method for evaluating EH in MD. However, it should be noted that EH could also occur in the healthy ear and various otological disorders. In addition, not all types of EH can be visualized in MRI [95]. Moreover, the spatial resolution of MRI could affect the interpretation. Advanced techniques and protocols to improve image acquisition and interpretation in the future would be helpful for clinicians to use MRI to predict EH in MD.

## 3. Molecular Markers for MD

### 3.1. Immunological/Autoimmunity Markers

Many studies have revealed that some autoimmune diseases were associated with MD. For instance, higher prevalences of systemic lupus erythematosus, ankylosing spondylitis or rheumatoid arthritis were observed in MD groups than in the general population [10,106]. Therefore, autoimmunity was thought as one of the possible causes of MD and the endolymphatic sac may play an important role in the immuno-mediated reaction. It has been speculated that one-third of MD causes seem to be of an autoimmune origin [107].

Several immunological markers were investigated to see whether they could differentiate MD patients from healthy controls. For example, heat shock proteins (HSPs) play an essential role in chaperoning functions, protein folding and protecting cells from physiological and ototoxic stresses [108]. The immunoglobulin G (IgG) antibodies to HSP70 (68-kD protein) were elevated in 30% of the MD group compared to 5% in the control group [109]. However, the results were varied in other reports, showing that 7.7% to 27% of MD cases were positive for HSP70 antibodies [110,111]. In fact, the detection of HSP70 antibodies in diagnosing MD is controversial because of the high prevalence of antiHSP70 antibodies in healthy subjects and the lack of association with disease activity [112].

Circulating immune complexes (CICs), the molecules comprising multiple antigens and antibodies, can damage targeted organs or tissues via complement activation with or without deposition to modulate the inflammation and immune reaction. In MD patients, some studies found elevated serum CICs and postulated that endolymphatic sac function was interfered with by CICs [113,114,115,116,117]. Additionally, increased total IgG, C3 and anti-type II collagen antibodies were found in MD [114,118,119,120].

Immunoglobulin E (IgE), induced by type I allergic reactions, is another immunological marker investigated in MD. It was reported that elevated total serum IgE was observed in patients in MD [116,121]. Interestingly, a recent study revealed that a high level of IgE was noted in patients with acute low-tone sudden sensorineural hearing loss, and a higher IgE level was correlated with an enhanced SP/AP ratio, which might be used as a predictor for MD [122]. In addition, Zhang et al. observed that IgE was correlated to the grading of EH, hearing stage and the functional level of MD patients [123], implying the association of allergy with the clinical severity of MD.

### 3.2. Inflammatory Markers

Since the immune system could be involved in the pathogenesis of MD, inflammatory responses may possibly occur in the inner ear [124]. As a result, several studies have investigated different cytokines and chemokines to see if they can be used as possible markers for MD. For example, Frejo et al. revealed that MD patients had higher basal levels of IL-1β, IL-1RA, IL-6 and TNF-α compared to healthy controls [125]. In addition, they observed the bimodal distribution of IL-1β levels in two different subgroups of MD, suggesting that a subset of MD patients have higher basal levels of proinflammatory cytokines. Later, they observed that patients with MD or VM have different proinflammatory signatures and concluded that the cytokine panel with IL- 1β, CCL3, CCL22,and CXCL1 levels might help to differentiate MD from VM [126].

The association of immunological and inflammatory markers with hearing loss and the stage of MD was recently investigated by Zhang et al. [127]. They checked CIC, HSP70 and TNF-α in the serum in MD patients with different hearing levels and staging. In addition to the increased concentration of these markers in MD, they observed that the phase of the pure tone average was positively associated with the concentration of CIC, HSP70 and TNF-α. In addition, the concentration of these markers was also increased in the group with severe EH compared to mild and moderate EH, implying that these immunological and inflammatory markers have the potential to reflect the EH severity and staging of MD.

### 3.3. Protein Signatures

The development of proteomics and protein array analysis in recent years has made it possible to survey possible protein markers in diseases. Kim et al. used Protoarray to investigate the proteins in sera from MD patients and controls. They observed higher signals of immunoglobulin heavy constant gamma 1 (IGHG1), the regulator of G-protein signaling 10 (RGS10), transcript variant 2, chromosome 2 open reading frame 34 (C2orf34), and SH3-domain GRB2-like endophilin B1 (SH3GLB1) with 80% sensitivity and specificity [128]. The authors imply that multiple antibodies or antigens might cause autoimmune reactions in the inner ear of MD.

In another study using proteomics to analyze the plasma from 15 MD patients and 12 healthy controls, upregulated complement factor-B and H, fibrinogen α-chain, β-actin, pigment epithelium-derived factor and fibrinogen γ-chain were found in MD, while vitamin D-binding protein, apolipoprotein A-1 and β-2-glycoprotein were downregulated compared to the control group [129]. They further used Western blotting to investigate plasma protein expression in different stages of MD patients [130]. They observed increased fibrinogen α- and γ-chain expression in stage III and decreased β-2-glycoprotein expression in stage IV patients. Stage I individuals have a higher expression of complement factor H and B proteins. They concluded that a set of plasma proteins might be used as a tool for a biomarker-oriented diagnosis and MD staging.

### 3.4. Vasopressin

Vasopressin, also known as antidiuretic hormone (ADH) or arginine vasopressin (AVP), is a nonapeptide that acts on water metabolism via vasopressin receptors. It was hypothesized that plasma vasopressin elevation and subsequent vasopressin type-2 receptor (V2R)-cyclic AMP (cAMP)-protein kinase A (PKA) activation in the endolymphatic sac might lead to the intracellular translocation of aquaporin-2 (AQP2) from the luminal side to the basolateral side with endosomal trapping, resulting in EH in the inner ear [131].

In previous decades, vasopressin has probably been the most investigated marker in MD. Takeda et al. first compared plasma vasopressin levels in patients with MD with other types of vertigo. They observed that vasopressin levels were higher in MD patients. In addition, vasopressin was significantly higher in the acute phase than remission phase [132]. Later on, Aoki et al. also showed increased vasopressin levels, osmolality and stress scores in the acute phase of MD [133,134]. However, there is no significant correlation between vasopressin levels, osmolality and stress score. Particularly, the patients with abnormally high vasopressin in the acute phase were resistant to conservative treatments for vertigo attacks [135]. They thus thought that the elevation of vasopressin in MD might be related to the pathogenesis of MD attacks.

In contrast, there are some studies showing conflicting results. Lim et al. compared the vasopressin levels of unilateral MD patients within one week of acute vertigo with 31 healthy volunteers, and they did not find statistical differences [136]. Similarly, Hornibrook et al. evaluated vasopressin levels in 80 patients with MD who were diagnosed using conventional symptoms and ECochG [137]. They still could not find the differences between the MD subjects and the normal controls. In addition, vasopressin levels did not correlate to the stage of MD. A recent meta-analysis revealed that the discrepancy of vasopressin levels between previous studies might be due to a couple of selection biases and confounding factors: unilateral vs. bilateral MD, acute vs. remission phase, the sensitivity of measurement and psychological stress might affect the levels of vasopressin [138].

Although there is no consensus regarding the usage of vasopressin as a diagnostic marker for MD, most publications agree that vasopressin might reflect the status of MD attack in MD patients [8], probably because the overexpression and hyperactivity of V2R in the endolymphatic sac of MD patients develop EH and vertigo attacks after vasopressin elevation [139]. A pilot study from Kitahara et al. observed that interventions to decrease vasopressin levels by adequate water intake, tympanic ventilation tube insertion and sleeping in darkness are useful to control MD [140]. Interestingly, the authors also found that in patients with intractable MD, vasopressin levels were reduced after endolymphatic sac surgery, and long-lasting plasma low vasopressin levels were associated with good surgical outcomes. Their findings implied that plasma vasopressin levels might be a feasible marker to reflect the disease status of MD [131].

### 3.5. Circadian Clock Genes

The circadian clock is present in eukaryotes with a 24 h cycle, and daily rhythmic changes can be observed in many physiological processes. In mammals, the circadian clock genes regulate circadian rhythms through transcriptional-translational feedback loops. Once the circadian clock is dysregulated or disrupted due to light changes, the expression of circadian clock genes is altered [141]. Evidence shows that circadian disruption is associated with an increased risk of several diseases, and altered circadian clock gene expression was frequently observed in patients with these disorders [142,143].

**Figure 1:**
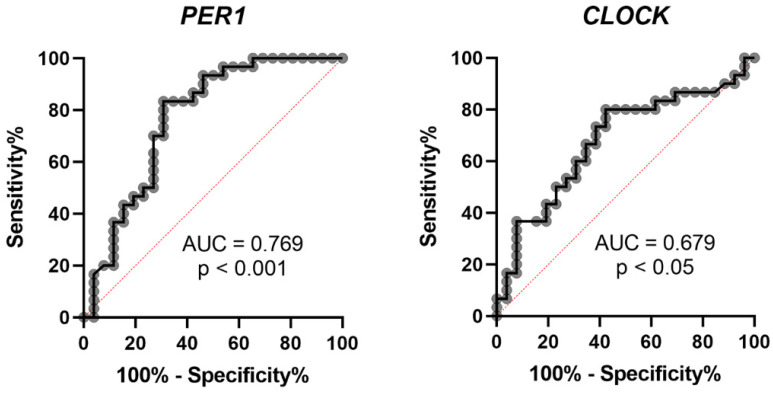
Receiver operating characteristic (ROC) curve of *PER1* and *CLOCK* to predict the diagnosis of MD. AUC: area under the curve.

Previous studies have proven that circadian clock genes have time-dependent variation patterns in the peripheral blood leukocytes of healthy subjects [142,144]. Because the precipitating factors for vertigo attacks in MD, such as a high-salt diet, caffeine and stress, might affect the circadian clock [145], we recently investigated the expression of circadian clock genes from the peripheral blood leukocytes of unilateral MD patients with recent vertigo attacks within one week to reflect the gene expression in the active status of MD [146]. We observed significantly decreased *PER1* and increased *CLOCK* gene expression in the MD group compared to a healthy control group using real-time quantitative reverse transcriptase-polymerase chain reaction (qRT-PCR) analysis. Particularly, the area under the receiver operating characteristic (ROC) curve (AUC) is higher in the *PER1* gene to predict the diagnosis of MD (Figure 1). Further immunocytochemical analysis for *PER1* in PB leukocytes also revealed the lower percentage of PER1-positive cells in the peripheral blood of MD patients [146]. These results implied that the expression of *PER1* might be a potential marker of MD.

Another implication of *PER1* as a marker of MD is to see if *PER1* expression is associated with disease severity. In the subgroup analyses of the circadian clock genes in groups with different dizziness handicaps and hearing levels, the expression of *PER1* was not different between the patients with mild-to-moderate and severe dizziness handicaps but is significantly lower in patients with stage 3 and 4 than with stage 1 and 2 [146]. The down expression of *PER1* was also significantly correlated to the pure tone average and speech reception threshold of the affected ear, implying that *PER1* might also be a potential marker for evaluating the hearing levels of MD patients.

The mechanism of altered *PER1* in the pathogenesis of MD and its effects on hearing levels still need further investigation. We hypothesized that since *PER1* in PB leukocytes may reflect the human circadian system [144], its dysregulation might affect cellular glutathione peroxidase-related reactive oxygen species fluctuation and augment oxidative stress in the hair cells [147]. In addition, our data were evaluated using MD patients in the acute phase, which was deemed to be a consequence of precipitating factors; it is reasonable to speculate that *PER1* expression may be different between the patients in acute and remission phases. Future exploration is necessary to evaluate the role of circadian clock genes as markers of MD.

### 3.6. The Problems and Future Directions of Molecular Markers for MD

Although the aforementioned molecular markers have been investigated based on the possible mechanisms of MD, such as autoimmunity, inflammation, hormone and circadian clock alteration, the design of most studies is cross-sectional. The reason is probably that the diagnosis of definite MD needs long-term follow-up, and longitudinal studies to evaluate the potential molecular markers are difficult. In addition, the expression of the molecular markers might be confounded by environmental factors. For example, plasma vasopressin levels might change due to stress and the circadian clock. Furthermore, different disease statuses, such as active vs. remission phase, unilateral MD vs. bilateral MD and the severity of EH and hearing loss might affect the results of these molecular markers. Therefore, a longitudinal design to evaluate targeted molecular markers and appropriately analyze the results by subgrouping MD patients would be mandatory to develop suitable molecular markers for MD. Last, since the specificity of the molecular markers from the peripheral blood may be limited by environmental factors, it would be more valuable to check the expression of targeted markers in the inner ear fluid (during the endolymphatic sac surgery) to accurately understand the role of these markers in the etiopathogenesis of MD [128].

In recent years, there has been a growing theory about the connection between migraine and MD. It was hypothesized that because of similar clinical manifestations, epidemiological factors and pathophysiological considerations [148], MD and migraine may be different regional manifestations of the same pathology. Another researcher also proposed that MD might be a “cochleovestibular migraine”, which resembles a combination of symptoms from cochlear and vestibular migraine [12]. If the hypothesis is true, the molecular markers for migraine and MD might be similar if the samples were gathered from the peripheral blood. Indeed, migraineurs had higher levels of serum inflammatory markers such as proinflammatory cytokines [149,150]. Increased plasma vasopressin levels are observed in migraine patients during an attack [151,152]. However, it is not clear whether the migraine biomarkers such as glutamate, calcitonin gene-related peptide (CGRP) and pituitary adenylate cyclase-activating peptide-38 (PACAP-38) are also markers of MD or can be used to differentiate migraine from MD [153]. The comparison of MD and migraine markers between the patients with MD and VM will help to elucidate the interplay between migraine and MD. In addition, since the diagnosis of MD and VM is based on the clinical diagnostic criteria, the development of a useful molecular marker to tell MD from VM would be a new direction toward the precise diagnosis of MD.

## 4. Conclusions and Future Perspectives

MD is a disease that is difficult to diagnose, particularly in the early stage wherein not all of the typical symptoms are present. The diagnosis of MD is based on the clinical diagnostic criteria. However, the development of functional and molecular markers for MD is important for helping clinicians diagnose MD correctly and evaluate inner ear status. Table 2 summarizes the markers for MD based on different aspects. For functional markers, ECochG and MRI have a relatively high sensitivity. Therefore, these tests could be used to confirm the EH in MD in ambiguous cases and those with atypical symptoms. The incidence of abnormalities in PTA, ECochG, VEMP, caloric test and MRI are higher in patients with high levels of hearing loss. Therefore, the positive results in these tests could reflect the advanced stages of MD. However, we shall keep in mind that the results of VEMP, caloric test and vHIT may be different in active and quiescence status. As a result, the abnormalities of these tests may reflect recent vertigo attacks. All functional markers might help clinicians to differentiate between MD and VM. For molecular markers, the majority of them might differentiate MD and healthy control as well as correlate to the hearing stage. However, further investigation is necessary to elucidate their expression between and during vertigo attacks and to know whether they are helpful in differentiating MD and VM. Looking ahead, although we have numerous papers to investigate the role of functional and molecular markers in MD, the duplication and verification of these markers are mandatory for proof of future usefulness in the clinic. There is still a long way to go to obtain ideal markers for MD right now, but we could still combine several markers to help diagnose and evaluate MD status. For instance, the combination of function markers, molecular markers, and 3D-FLAIR MRI may be a better strategy for helping in the diagnosis and evaluation of MD. However, before a perfect protocol of markers is developed, clinicians shall keep in mind that it is still essential to diagnose MD using the latest diagnostic criteria.

## Figures and Tables

**Table 1:** Diagnostic criteria of MD according to 2015 AAO-HNS Equilibrium Committee [17].

**Definite MD:**
1. Two or more spontaneous attacks of vertigo, each lasting 20 min to 12 h
2. Audiometrically documented fluctuating low- to midfrequency sensorineural hearing loss in the affected ear on at least 1 occasion before, during, or after 1 of the episodes of vertigo
3. Fluctuating aural symptoms (hearing loss, tinnitus, or fullness) in the affected ear
4. Other causes excluded by other tests
**Probable MD:**
1. At least 2 episodes of vertigo or dizziness lasting 20 min to 24 h
2. Fluctuating aural symptoms (hearing loss, tinnitus, or fullness) in the affected ear
3. Other causes excluded by other tests

**Table 2:** Summary of functional and molecular markers for MD.

	Differentiate MD From Healthy Controls	Differentiate Hearing Levels and Stages of MD	Differentiate Vertigo Attacks with Remission in MD	Differentiate MD from VM
**Functional** **markers**				
PTA	Documented audiometry is mandatory to diagnose definite MD	Stages of MD are defined by the hearing levels of the affected ear	The fluctuated hearing loss is not always related to vertigo attacks [31]	Typical MD exhibits low-tone hearing loss with progression. VM usually could recover from low-tone hearing loss [22,25].
ECochG	Sensitivity: 66.7–85.7% Specificity: 80–100% [36]	Increased SP/AP ratio in patients with higher levels of hearing loss [42,48,49] and longer duration of the disease [38]	SP/AP ratio does not recover even if vertigo attacks disappear [38]	A higher proportion of abnormal ECochG in MD than VM [50]
VEMP	Sensitivity: 49% Specificity: 95% [58]	IAD ratio of VEMP increased in the advanced stage of MD [57]	Differ between quiescence and acute attack status [64]	MD group showed a reduction in tone-evoked amplitudes for oVEMP [65] and the prevalence of a higher IAD ratio compared to the VM group [66]
Caloric test/vHIT	47–67% of patients with MD have unilateral canal weakness [70,71]. The incidence of vHIT abnormality is lower than the caloric test [78]	The incidence of canal paresis in the caloric test is higher in the advanced stage of MD [73]. No differences in abnormal vHIT results between different stages of MD [73,76].	Caloric responses are usually diminished during the attacks of MD [72] vHIT results may differ during and between acute vertigo attacks [72,74,75]	Incidences of abnormal caloric test and vHIT are higher in MD than in VM [78]
MRI	sensitivity: 79.5–84.6% specificity: 92.3–93.6% [85]	MRI EH degree has a positive correlation between the hearing level and the vestibular EH degree [87,88,89,90]	The grade of EH is not correlated with the extent of vertigo [87,89]. EH is stable during and after vertigo attacks [93].	A higher incidence of EH was observed in MD compared to VM [102,103]
**Molecular** **markers**				
Immunological/ autoimmunity markers	Increased HSP70 antibodies [109], CICs [117] and IgE [123] in MD	The phase of pure tone average was positively associated with HSP70 and CIC [127] IgE was correlated to the hearing stage [123]	Not determined	Not determined
Inflammatory markers	MD patients had higher basal level of IL-1β, IL-1RA, IL-6 and TNF-α compaed to healthy controls [125]	The phase of pure tone average was positively associated with TNF-α [127]	Not determined	Cytokine panel with IL- 1β, CCL3, CCL22, and CXCL1 levels may help to differentiate the MD from VM [126].
Protein signatures	Higher several protein signatures in MD [128,129,130]	Increased fibrinogen α- and γ-chain expression in stage III and decreased β-2-glycoprotein expression in stage IV patients. Stage I individuals have a higher expression of complement factor H and B proteins [130]	Not determined	Not determined
Vasopressin	Vasopressin levels were higher in MD patients [132,133,134,135] or no difference between MD and controls [136,137]	Vasopressin levels did not correlate with the disease stage of MD [137]	Vasopressin levels were significantly higher in the acute phase than remission phase [132]	Not determined
Circadian clock genes	Decreased *PER1* and increased *CLOCK* gene expression in the MD group compared to a healthy control group [146]	*PER1 is* significantly lower in patients with stage 3 and 4 compared to stage 1 and 2 [146] Down expression of PER1 was significantly correlated to the pure tone average and speech reception threshold of the affected ear [146]	Not determined	Not determined

MD: Meniere’s disease; VM: vestibular migraine; IAD: interaural amplitude difference; ECochG: electrocochleography; VEMP: vestibular evoked myogenic potential; vHIT: video head impulse test; MRI: magnetic resonance imaging; HSP: heat shock protein; CIC: circulating immune complex.

## Data Availability

Data are available to the corresponding authors upon request.
